# DEAD-Box Protein RNA-Helicase DDX6 Regulates the Expression of HER2 and FGFR2 at the Post-Transcriptional Step in Gastric Cancer Cells

**DOI:** 10.3390/ijms19072005

**Published:** 2018-07-09

**Authors:** Toshihiro Tajirika, Yoshihisa Tokumaru, Kohei Taniguchi, Nobuhiko Sugito, Nobuhisa Matsuhashi, Manabu Futamura, Kazuyoshi Yanagihara, Yukihiro Akao, Kazuhiro Yoshida

**Affiliations:** 1 Department of Surgical Oncology, Graduate School of Medicine, Gifu University, 1-1 Yanagido, Gifu 501-1194, Japan; t-tazi@live.jp (T.T.); yoshitoku1090@gmail.com (Y.T.); nobuhisa@gifu-u.ac.jp (N.M.); mfutamur@gifu-u.ac.jp (M.F.); kyoshida@gifu-u.ac.jp (K.Y.); 2 Department of General and Gastroenterological Surgery, Osaka Medical College, 2-7 Daigaku-machi, Takatsuki, Osaka 569-8686, Japan; sur144@osaka-med.ac.jp; 3 Translational Research Program Osaka Medical College, 2-7 Daigaku-machi, Takatsuki, Osaka 569-8686, Japan; 4 United Graduate School of Drug Discovery and Medical Information Sciences, Gifu University, 1-1 Yanagido, Gifu 501-1193, Japan; v3501002@edu.gifu-u.ac.jp; 5 Division of Biomarker Discovery, Exploratory Oncology and Clinical Trial Center, National Cancer Center, 6-5-1 Kashiwa, Chiba 277-8577, Japan; kyanagih@east.ncc.go.jp

**Keywords:** RNA-helicase DDX6, FGFR2, HER2, gastric cancer

## Abstract

The human DEAD/H-box RNA helicase DDX6 (RCK/p54) is a protein encoded by the fusion gene from the t(11;14)(q23;q32) chromosomal translocation observed in human B-cell lymphoma cell line RC-K8. DDX6 has a variety of functions such as translation initiation, pre-mRNA splicing, and ribosome assembly. However, details of the regulatory mechanism governing DDX6 and the functions of DDX6 are largely unknown. Previously, we reported that DDX6 is overexpressed in most malignant cell lines and clinical colorectal tumor samples and that DDX6 positively contributes to the pathogenesis of various cancers. In the current study, we aimed at revealing the function of DDX6 in HER2 and FGFR2 related human gastric cancer (GC) by using clinical samples and GC cell lines. DDX6 protein was overexpressed in about 60% of the clinical samples; HER2, in 35%; and FGFR2, in 30%, (*n* = 20). Interestingly, the DDX6 protein was overexpressed in all HER2-positive samples (*n* = 7), and in 83% (5 of 6) of the FGFR2-positive samples, which could reflect the contribution of DDX6 to the expression of HER2 and FGFR2. In the GC cell line MKN7, which has *HER2* amplification, the knockdown of *DDX6* by siR-DDX6 led to the decreased expression of the HER2 protein. On the other hand, the knockdown of *HER2* did not influence the DDX6 expression. Similar results were also obtained for the KATO-III and HSC39 cell lines having amplified FGFR2 expression. The increased expression of DDX6 induced a significantly increased expression of the HER2 protein without increasing the mRNA expression. The results of an RNP Immunoprecipitation (RIP)-assay using GC cells indicated that the DDX6 protein acted as an RNA-binding protein for *HER2* and FGFR2 mRNAs and positively regulated their post-transcriptional processes. These findings demonstrated that DDX6 was an upstream molecule that positively regulated the expression of HER2 and FGFR2 at the post-transcriptional step in GC cells.

## 1. Introduction

Gastric cancer (GC) is the fifth most common cancer and third most common cause of cancer-related deaths worldwide. More than 720,000 deaths are attributed to GC annually [1]. Nowadays, GC is diagnosed in its early stages owing to improvements in endoscopy. However, many cases are still first found in the advanced stage [1,2]. Therefore, the efficacy of chemotherapy determines their prognosis. In chemotherapy against GC, we use traditional drugs, such as 5-fluorouracil, cisplatin, and taxans, as well as molecular targeting drugs, such as trastuzumab (an anti-human epidermal growth factor receptor 2 (HER2) antibody) and ramucirumab (the anti-vascular endothelial growth factor receptor 2 (VEGFR2) monoclonal antibody). Recently, an immune checkpoint inhibitor, nivolumab, has become available [3]. However, advanced GC is still considered to be a fatal disease. The efficacy of chemotherapy for advanced or recurrent GC is not satisfactory because the median survival time of patients having undergone chemotherapy without an immune checkpoint inhibitor is reported to be only 6 to 13 months in some trials [4]. Thus, there is a need for the identification of new therapeutic target molecules and novel biomarkers for individualized therapy and early diagnosis. Nowadays, details about the genetic abnormalities in human GC, such as mutations, amplifications, and rearrangements, are being revealed [5]. However, no molecular targets for GC therapy have yet been suggested.

In such a situation, we tried to clarify what mechanism works in the pathogenesis involving the gene abnormalities of *HER2*, *KRAS*, *FGFR2*, *MET*, *EGFR*, *PI3K*, *BRAF*, and so on in GC. Interestingly, the amplifications of such genes are observed frequently (41%) in GC [5].

DDX6 (also termed RCK/p54) belongs to the family of human DEAD/H-box RNA helicases, and more from the t(11;14)(q23;q32) chromosomal translocation observed in human B-cell lymphoma cell line RC-K8. DDX6 has many functions such as translation initiation, pre-mRNA splicing, ribosome assembly by acting as an RNA-binding protein. Additionally, DDX6 contributes to the proliferation and differentiation in the stem and progenitor cells [6]. However, details regarding the mechanism of action and functions of DDX6 are largely unknown.

Previously, we reported that the DDX6 is overexpressed in most malignant cell lines and clinical colorectal tumor samples examined [7], and that DDX6 positively contributes to c-Myc expression at the translation initiation step in various cancers [8]. In the current study, we sought to clarify the role of DDX6 in the overexpression of HER2 and FGFR2 seen in GC cells.

## 2. Results

### 2.1. Expression Levels of DDX6, HER2, and FGFR2 in GC Clinical Tumor Samples

**Figure 1:**
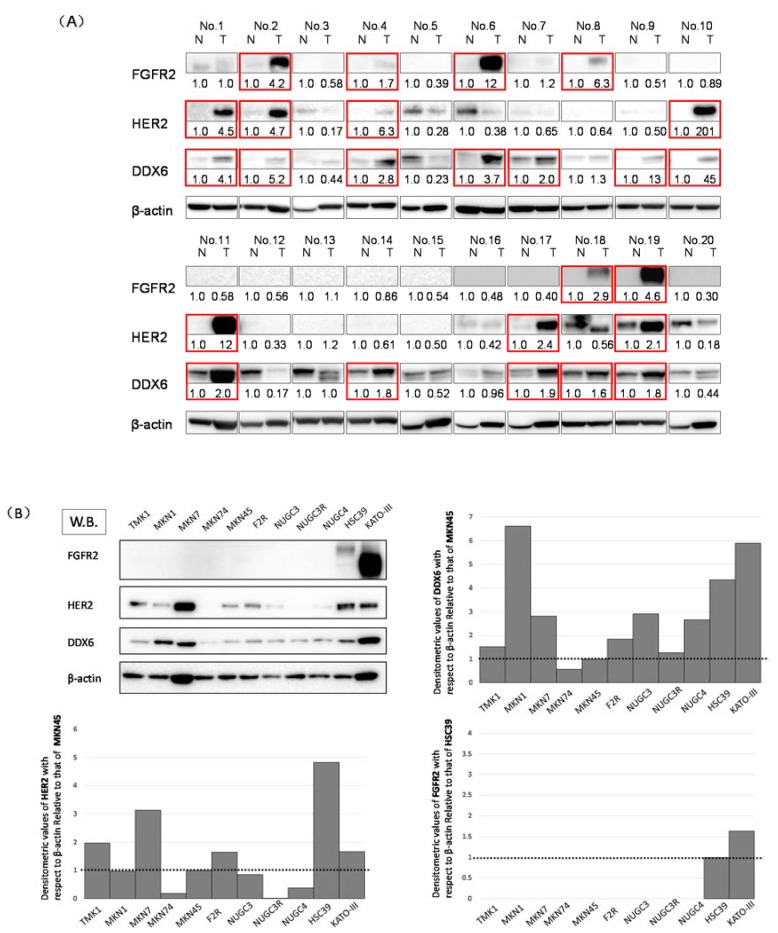
The expression levels of DDX6, HER2, and FGFR2 in gastric cancer (GC) cell lines and GC clinical samples. (**A**) Western blots of the 20 clinical samples. The overexpression of DDX6, HER2, and FGFR2 in tumor samples is highlighted by red-boxes. N: normal, T: tumor in the same patient. Details of the characteristics of the patients are given in Table 1. Densitometric values of DDX6, HER2, and FGFR2 with respect to β-actin of each sample were calculated and the values are shown with each normal sample taken as 1.0. Densitometric values more than 1.5 are regarded as overexpressed. The cases that overexpressed DDX6, HER2, and FGFR2 are highlighted by red boxes; (**B**) Western blotting (W.B.) of the steady-state levels of DDX6, HER2, and FGFR2 in GC cell lines. Cell lines including TMK1, MKN1, MKN7, MKN74, MKN45, F2R (MKN45-5FU resistant [5FUR]), NUGC3, NUGC3-5FUR, NUGC4, HSC39, and KATO-III. In the expression levels of HER2 and DDX6, densitometric values of DDX6 with respect to β-actin of each sample were calculated and the values are shown with MKN45 taken as 1.0. In the case of FGFR2, the values are shown with HSC39 taken as 1.0.

First, we used Western blotting to investigate the expression levels of DDX6, HER2, and FGFR2 in 20 GC clinical tumor samples. The expression levels of DDX6, HER2, and FGFR2 in these samples were increased in 12 (60%), 7 (35%), and 6 (30%) samples, respectively; 9 of 10 (90%) patients with the high expression levels of FGFR2 and/or HER2 also showed DDX6 overexpression (Figure 1B). These results suggested to us that RNA helicase DDX6 may be related to the expression of HER2 and FGFR2.

### 2.2. Expression Levels of DDX6, HER2, and FGFR2 in GC Cell Lines

We also investigated the expression levels of DDX6, HER2, and FGFR2 in TMK1, MKN7, MKN74, MKN45, F2R, NUGC3, NUGC3/5-FU, NUGC4, HSC39, and KATO-III cells by using Western blotting (Figure 1A). The expression levels of FGFR2 in HSC39 and KATO-III cells, which have *FGFR2* gene amplification, were increased compared with those of the other cell lines tested. Additionally, the expression levels of HER2 in MKN7 cells, which amplified the expression of the *HER2* gene, and those in the HSC39 and KATO-III cells, were increased compared with those of the other cell lines. Interestingly, the expression levels of DDX6 were significantly increased in MKN1, MKN7, HSC39, and KATO-III cells, which overexpressed FGFR2 and/or HER2. We selected the MKN7, MKN45, KATO-III, and HSC39 cell lines because HER2 and/or FGFR2 amplification and their overexpression were observed in these cell lines. MKN45 that expresses low levels of DDX6 and HER2 proteins was used as a control cell line (Figure 1B).

### 2.3. Effect of Knockdown of DDX6 on Expression of FGFR2 and HER2 in MKN7, MKN45, HSC39, and KATO-III Cells

**Figure 2:**
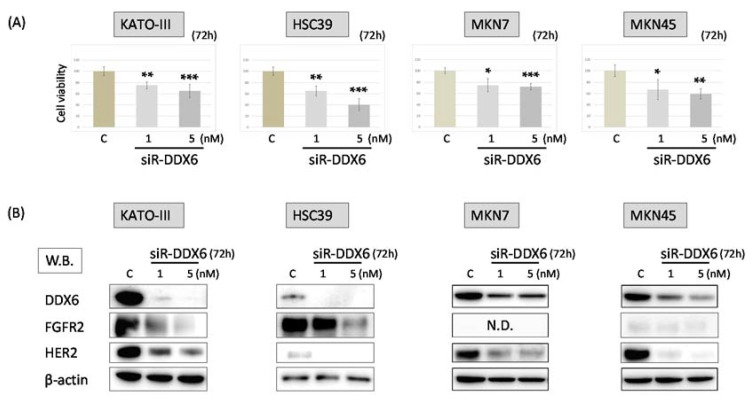
The knockdown of *DDX6* in MKN7, MKN45, HSC39, and KATO-III cells by siRNA treatment. (**A**) Cell viability at 72 h after transfection of KATO-III, HSC39, MKN7, and MKN45 with siR-DDX6. C: control RNA, 5 nM. Controls are indicated as “100”; (**B**) Western blots of FGFR2, HER2, and DDX6 expression in KATO-III, HSC39, MKN7, and MKN45 cells at 72 h after transfection with control or siR-DDX6. The levels of controls are indicated as “1”. N.D.: not detected. Results are presented as the mean ± SD. * *p* < 0.05; ** *p* < 0.01; *** *p* < 0.001.

In order to elucidate the relationship between DDX6 and the expression of HER2 and FGFR2, we examined the cell viability of MKN7, MKN45, HSC39, and KATO-III cells and the expression of FGFR2 and HER2 in them after the knockdown of *DDX6* by use of siRNA for *DDX6* (siR-DDX6). The number of viable cells in all cell lines tested was significantly reduced at 72 h after post transfection, even at the concentration of 1 nM siR-DDX6 (Figure 2A). Additionally, the knockdown of *DDX6* led to decreased expression levels of HER2 and FGFR2 in these cells. These results indicated that DDX6 positively regulated the expression of HER2 and FGFR2 (Figure 2B). 

### 2.4. DDX6 Expression after the Knockdown of FGFR2 in HSC39 and KATO-III Cells

**Figure 3:**
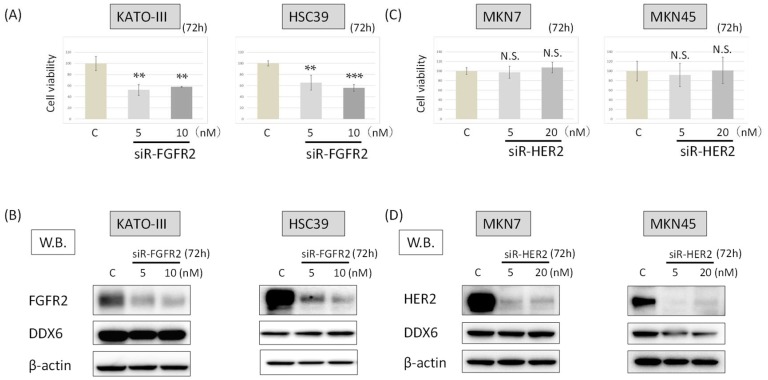
The DDX6 expression after the knockdown of *FGFR2* or *HER2*. (**A**) Cell viability at 72 h after transfection of KATO-III and HSC39 cells with siR-FGFR2. (control RNA, 10 nM); (**B**) Western blots of FGFR2 and DX6 expression in KATO-III and HSC39 cells at 72 h after transfection with control or siR-FGFR2; (**C**) Cell viability at 72 h after transfection of MKN7 and MKN45 with siR-HER2. (control RNA, 20 nM); (**D**) Western blots of HER2 and DDX6 expression in MKN7 and MKN45 cells at 72 h after transfection with control or siR-HER2. The levels of controls are indicated as “1.” Results are presented as the mean ±SD. ** *p* < 0.01; *** *p* < 0.001. N.S., not significant.

Next, we examined the growth of HSC39 and KATO-III cells and their expression of DDX6 at 72 h after *FGFR2* silencing (siR-FGFR2), the cell viability for both cell types was significantly reduced to about 40–50% of the control (Figure 3A). On the other hand, the knockdown of *FGFR2* did not change the expression levels of DDX6 in HSC39 or KATO-III cells (Figure 3B). These results suggested that DDX6 acted upstream of *FGFR2* to regulate the FGFR2 expression.

### 2.5. DDX6 Expression after the Knockdown of HER2 in MKN7 and MKN45 Cells

We also examined whether knockdown of *HER2* with siRNA for HER2 (siR-HER2) would influence the expression level of DDX6 in and viability of MKN7 and MKN45 cells. The numbers of viable cells remained almost unchanged compared with that of the control cells at 72 h after the knockdown of *HER2* (Figure 3C). Additionally, the knockdown of *HER2* did not affect the expression levels of DDX6 in either cell type (Figure 3D).

These results indicated that DDX6 also functioned upstream of *HER2* to regulate the expression of HER2.

### 2.6. HER2 and FGFR2 mRNA Levels after Knockdown of DDX6 in MKN7, MKN45, HSC39, and KATO-III Cells

**Figure 4:**
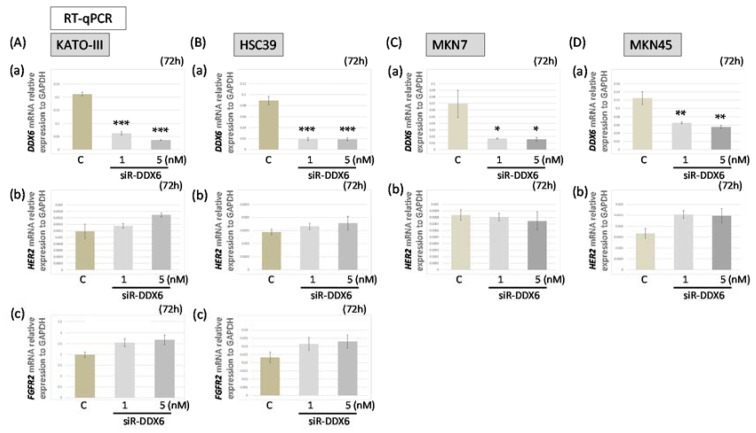
The *HER2* and *FGFR2* mRNA levels in MKN7, MKN45, HSC39, and KATO-III cells after knockdown of *DDX6* in the cells. (**A**,**B**) The expression levels of *DDX6* (a), *HER2* (b), and *FGFR2* (c) mRNAs at 72 h after transfection of KATO-III (**A**) and HSC39 (**B**) cells with siR-DDX6; (**C**): control RNA, 5 nM; (**C**,**D**) Expression levels of *DDX6* (**a**) and *HER2* (**b**) mRNAs at 72 h after transfection of MKN7 (**C**) and MKN45 (**D**) cells with siR-DDX6. (C: control RNA, 5 nM) The levels of controls are indicated as “1.” Results are presented as the mean ± SD. * *p* < 0.05; ** *p* < 0.01; *** *p* < 0.001.

Furthermore, we used real-time RT-PCR to examine the mRNA levels of *HER2*, *FGFR2*, and *DDX6* in siR-DDX6-transfected MKN7, MKN45, HSC39, and KATO-III cells (Figure 4). We confirmed that the expression level of *DDX6* mRNA was extremely decreased in all siR-DDX6-treated cells (Figure 4A–D). Interestingly, the levels of *HER2* and *FGFR2* mRNAs in *DDX6*-silenced cells were slightly increased in a concentration-dependent manner compared with those of the control, whereas the protein expression levels of both FGFR2 and HER2 were significantly down-regulated in KATO-III and HSC39 cells (Figure 2B). Additionally, similar expression profiles were observed in the cases of MKN7 and MKN45 cell lines (Figure 2B and Figure 4C,D). These results suggested that DDX6 acted at a post-transcriptional step for the expression of HER2 and FGFR2.

### 2.7. RNA Immunoprecipitation (RIP)-Assay Using GC Cells

**Figure 5:**
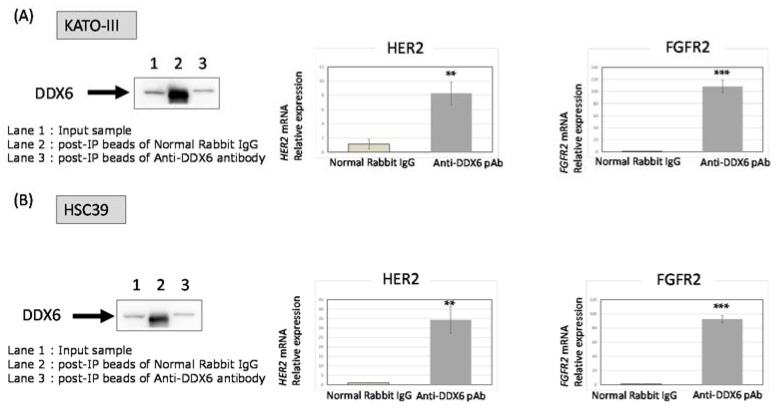
The RIP-assay for KATO-III and HSC39 cells by use of the DDX6 antibody. Immunoprecipitants formed by the anti-DDX6 antibody in the case of KATO-III (**A**) and HSC39 (**B**) cells were evaluated by W.B. for DDX6. Lane 1: Input sample; Lane 2: post-IP beads with normal rabbit IgG; Lane 3: post-IP beads with the anti-DDX6 antibody. Anti-rabbit IgG was used as a negative control. The expression levels of *HER2* (**left**) and *FGFR2* (**right**) mRNA in DDX6-RNA complexes were evaluated by RT-PCR in KATO-III (**A**) and HSC39 (**B**) cells. The levels of controls are indicated as “1”. The results are presented as the mean ± SD. ** *p* < 0.01; *** *p* < 0.001.

In order to validate the role of DDX6 in the translational step of HER2 and FGFR2 expression, we performed a RIP-assay. Its results clearly indicated that the DDX6 protein acted as an RNA-binding protein for *HER2* and *FGFR2* mRNAs and it positively regulated the translational process (Figure 5).

## 3. Discussion

**Figure 6:**
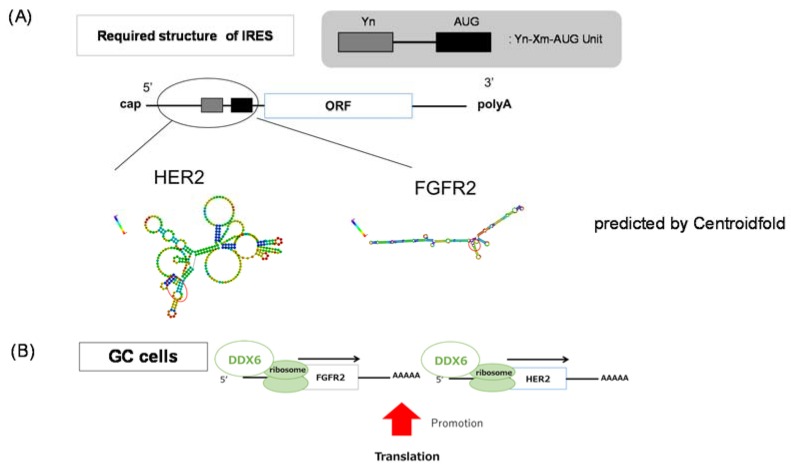
Predicted IRES structure of the *HER2* and *FGFR2* mRNAs. (**A**) IRES structure in the 5′ UTR of *HER2* and *FGFR2* mRNAs as predicted by Centroidfold (http://rtools.cbrc.jp/centroidfold/); (**B**) The scheme of DDX6 regulation of HER2 and FGFR2 in the translational step.

Previously, we reported that oncogenic RNA helicase DDX6 was highly overexpressed in most of the malignant cell lines and clinical colorectal tumors that we had tested [7,8]. We also showed that DDX6 expression is linked to the regulation of translation of *c-myc* mRNA through the internal ribosome entry site (IRES) [9]. In addition, the DDX6 protein is most likely to interact with the 5-cap-structure binding protein eIF4E [6,10], which is a rate-limiting factor serving as a translation regulator for initiating translation. In GC, more than 50% of the clinical GC samples we examined showed the increased expression of DDX6 compared with the normal samples from the same patients. Most of the DDX6-overexpressed samples also showed HER2 and/or FGFR2 overexpression, as estimated by Western blot analysis. Similar findings were also made for the cell lines tested. Based on such findings, we speculated that DDX6 might be involved in the overexpression of HER2 and FGFR2 proteins, which is due to the gene amplifications observed in GC. Thus, we determined that DDX6 was positively associated with translational initiation for *HER2* and *FGFR2* mRNAs, validated by the results of the RIP-assay and by the experiments using siRNAs for *DDX6*, *HER2*, and *FGFR2* (Figure 4, Figure 5 and Figure 6). We also found the feedback system of the DDX6-HER2 and DDX6-FGFR2 translational process because the down-regulation of HER2 and FGFR2 protein expression by silencing DDX6 induced the up-regulation of their mRNA levels. It is clear that DDX6 contributed to the maintenance of overexpression of HER2 and FGFR2 even in the presence of genetic aberrations. However, when we introduced the DDX6 expression vector into MKN74, which barely expressed HER2 and FGFR2, their protein expression levels were not increased (data not shown). DDX6 might work to efficiently express the amplified *HER2* and *FGFR2*. It is speculated that DDX6 is necessary to maintain the higher protein levels of these amplified *HER2* and *FGFR2*. RIP-assay showed that the DDX6 protein directly or indirectly influences the post-transcriptional step of *HER2* and *FGFR2*. High-throughput sequencing of RNA isolated by crosslinking immunoprecipitation (HITS-CLIP) or photoactivatable-ribonucleoside-enhanced crosslinking and immunoprecipitation (PAR-CLIP) may directly indicate the RBP binding motif.

HER2 belongs to the EGFR family and plays a key role in cell survival and proliferation [11,12]. Amplification of the *HER2* gene causes the overexpression of the HER2 protein, which causes cancer cell survival, growth, and proliferation through the activation of the *PI3K-AKT* and *MAPK-ERK* pathways [13,14]. HER2 overexpression is observed to range from 9 to 38% of study samples, depending on the location of the cancers and their histology [13,15,16,17,18,19]. In our study, the frequency of overexpression of HER2 was almost 35%, being in the higher part of the range. The role of HER2 as a prognostic biomarker of GC is still controversial. Some studies have reported a positive correlation between HER2 overexpression and the clinical features of the tumor, such as tumor size, lymph node metastasis, local invasion, and cancer stage; whereas other studies insisted no relationship between them [20,21,22,23,24,25,26]. HER2 overexpression has become a very important biomarker because the ToGa clinical phase 3 trial indicated that HER2-positive patients can get survival benefits by treatment with trastuzumab [27].

The *FGFR2* gene at human chromosome 10q26 encodes FGFR2b and FGFR2c isoforms that function as FGF receptors by separate expression domains and ligand specificity. Overexpression of FGFR2 was reported to be oncogenic and associated with chemoresistant in tumor cells. Single-nucleotide polymorphisms (SNPs) within intron 2 of the *FGFR2* gene are associated with breast cancer through allelic *FGFR2* upregulation [28]. A missense mutation or increased copy number of the *FGFR2* gene is present in breast cancer and GC to activate FGFR2 signaling. It is reported that an FGFR2 copy number gain is detected in 9% of whole cancers. In GC, *FGFR2* gene amplification ranges from 4 to 10% of samples; and FGFR2 protein is overexpressed in 30–40% of cases as judged by immunohistochemistry (IHC) [29,30]. When we estimated the expression of the FGFR2 protein by Western blotting, the rate of FGFR2 overexpression was almost 30%, similar to that observed in other studies. One important question is to ask what character of mRNA does DDX6 specifically affects in the translation initiation step. We considered that DDX6 might recognize the IRES structure of the 3’ UTR of mRNAs, serving as an RNA-binding protein (RBP) at the IRES structure by binding motifs of DDX6, possibly its N-terminal motif Ia and Ib, and C-terminal IV and V. It is reported that the IRES requires an oligopyrimidine and AUG unit structure (Figure 6A) [31]. We examined whether *HER2* and *FGFR2* have such an IRES structure by conducting a Centroidfold scan. Centroidfold is a web-available software that can predict and visualize RNA 2D structure (http://rtools.cbrc.jp/centroidfold/). As a result, we predicted that *HER2* and *FGFR2* might have the IRES structure (Figure 6A). Our results indicated that DDX6 RNA helicase promoted the translation of *FGFR2* and *HER2* mRNAs, which are frequently mutated or over-expressed in GC cells (Figure 6B), and functioned upstream of their expression pathways.

## 4. Materials and Methods

### 4.1. Clinical Samples

All clinical samples were obtained from patients who had undergone surgery for resection at the Department of Surgical Oncology, Gifu University Hospital (Gifu, Japan). Whether the tissue was tumorous or not was determined by the surgeon. All samples were immediately snap-frozen in liquid nitrogen and stored at −80 °C until RNA or protein extraction could be performed. All samples were histopathologically confirmed by H&E staining. The pathologic tumor staging was determined according to the Japanese Gastric Cancer Association (2011; see Table 1).

This study and collection and distribution of the samples were conducted in accordance with the principles of the 1975 Declaration of Helsinki after receiving approval from the Institutional Review Board of the Gifu University Graduate School of Medicine. (Approval number: 28–508 (23 March 2017)).

### 4.2. Cell Culture and Cell Viability

The TMK1, MKN1, MKN7, MKN45, MKN74, NUGC3, NUGC4, and KATO-III cells were obtained from the JCRB (Japanese Collection of Research Bioresources, Tokyo, Japan) Cell Bank. They were cultured in RPMI-1640 medium supplemented with 10% (*v*/*v*) heat-inactivated fetal bovine serum (FBS, Sigma-Aldrich Co., St. Louis, MO, USA) and 2 mm l-glutamine under an atmosphere of 95% air and 5% CO_2_ at 37 °C. The number of viable cells was determined by performing the trypan-blue dye-exclusion test. F2R, NUGC3 (5-FUresistance), and HSC39 were established cell lines [32,33,34].

### 4.3. Transfection Experiments

MKN7, MKN45, HSC39, and KATO-III cells were seeded into 6-well plates at a concentration of 0.5 × 10^5^ per well (10–30% confluence) on the day before the transfection. The sequence of siR-DDX6 was the following:

5′-GGAUAUUAUUCUCACGCUACCUAAA-3′. siR-HER2 and siR-FGFR2 were obtained from Invitrogen. They were used for the transfection of the cells, which was achieved by using cationic liposomes and Lipofectamine RNAiMAX (Invitrogen, Carlsbad, CA, USA), according to the manufacturer’s Lipofection protocol. The nonspecific control RNA (HSS, Hokkaido, Japan) sequence was: 5’-GUAGGAGUAGUGAAAGGCC-3’, which was used as a control for nonspecific effects [35].

### 4.4. Western Blot Analysis

Protein extraction and Western blot analysis were performed as described in previous reports [36,37]. The following primary antibodies were used: anti-FGFR2 and anti-HER2 (Cell Signaling Technology, Inc., Danvers, MA, USA), anti-DDX6 (RCK) (Santa Cruz Biotechnology (Dallas, TX, USA); sc-376433 K1414), and anti-β-actin (Sigma-Aldrich, St. Louis, MO, USA). HRP-conjugated goat anti-rabbit and horse anti-mouse IgG (Cell Signaling Technology, Danvers, MA, USA) were used as secondary antibodies. β-actin served as an internal control.

We used the web-available soft Image J in the case of calculating densitometric values.

### 4.5. Real-Time Reverse Transcription PCR

Total RNA was isolated from cultured cells by using a NucleoSpin (TaKaRa, Otsu, Japan 740955). For determination of the expression levels of DDX6, HER2, FGFR2, and GAPDH mRNAs, total RNA was reverse-transcribed with a PrimeScriptH RT reagent Kit (TaKaRa). RT-PCR was then performed with primers specific for them by using THUNDERBIRD SYBR qPCR Mix (TOYOBO, Osaka, Japan). The primers for *DDX6*, *HER2*, *FGFR2*, *c-Myc*, and *GAPDH* were the following:

*DDX6*-sense, 5′-GACACAGCAACAGATGAACC-3′ and *DDX6*-antisense, 5′-TGTCCTTCTT CAGGTCTAGC-3′ *HER2*-sense, 5′-CTGATGGGTTAATGAGCAAACTGA-3′ and *HER2*-antisense, 5′-CCAAATTCTGTGCTGGAGGTAGAG-3′ *FGFR2*-sense, 5′-AGTGCCCTCCCAGAGACCAA-3′ and *FGFR2*-antisense, 5′-CCCAGTTTCTCAATGAAGCCATAAA-3′ *GAPDH*-sense, 5′-CCACCCATGG CAAATTCCATGGCA-3′ and *GAPDH*-antisense, 5′-TCTAGACGGCAGGTCAGGTCCACC-3′.

### 4.6. RIP-Assay

The RIP-assay was performed by using a RIP-Assay kit (MBL, MEDICAL and BIOLOGICAL LABORATORIES CO., Nagoya, Japan). Cell lysates were derived from treated cells and reacted with beads labeled with the anti-DDX6 antibody. The target protein bound to the beads was then isolated, and the mRNAs binding the target protein were measured by qRT-PCR following the manufacturer’s procedures.

### 4.7. Statistics

Each examination was performed in triplicate. Statistical significances of differences were evaluated by performing the two-sided Student’s *t*-test. The values were presented as the mean ± standard deviation. A *p*-value < 0.05 was considered to be statistically significant.

## 5. Conclusions

Our results afforded the conclusion that DDX6 acted as an upstream molecule that positively regulated the expression of HER2 and FGFR2 at the post-transcriptional step in GC cells. Therefore, a DDX6 inhibitor such as a low-molecular agent and a siRNA targeting DDX6 would be promising drugs for HER2 and FGFR2-positive GCs.

## Figures and Tables

**Table 1:** Patient characteristics.

Case	Age	Sex	Site	Form	Pathological Findings	UICC/TNM	T	N	M	Stage
1	71	M	L	Type3	tub2	□	T3	N0	M0	IIA
2	91	F	L	Type2	tub2	□	T4b	N2	M0	IIIC
3	72	M	L	Type3	tub1	□	T4a	N3a	M0	IIIC
4	61	M	L	Type2	tub2	□	T4a	N3a	M0	IIIC
5	83	M	L	Type2	por1	□	T3	N0	M0	IIA
6	77	M	UM	Type3	muc > tub2	□	T3	N1	M0	IIB
7	66	F	LM	Type1	tub2	□	T2	N2	M0	IIB
8	84	M	MLD	Type3	tub2	□	T4a	N3a	M0	IIIC
9	85	F	M	Type2	por2 > tub1	□	T3	N0	M0	IIA
10	72	M	ML	Type1	tub1 > pap	□	T1b	N0	M0	IA
11	64	M	U	Type1	tub2	□	T3	N0	M0	IIA
12	78	M	LM	Type2	por1	□	T4b	N3a	M0	IIIC
13	64	F	GE	Type2	pap > muc	□	T3	N0	M0	IIA
14	73	M	M	Type1	tub1 (>tub2)	□	T1a	N0	M0	IA
15	85	F	M	Type3	por1 > tub1	□	T2	N2	M0	IIB
16	73	M	LMU	Type4	por2 > muc	□	T4a	N3a	M0	IIIC
17	80	M	UE	Type2	tub2	□	T3	N0	M0	IIA
18	93	M	ML	Type3	tub2	□	T4a	N0	M0	IIB
19	65	F	L	Type2	tub2 > tub1	□	T2	N0	M0	IB
20	74	F	U	Type3	por2 > sig > muc	□	T4a	N3a	M0	IIIC

SEX; M, male; F, female. Site; Location of tumor; U, upper; M, middle; L, lower; R, residual gastric cancer. Form; Macroscopic classification: Type 1, mass type; Type 2, localized ulcerative type; Type 3, infiltrative ulcerative type; Type 4, diffuse infiltrating type; Type 5, unclassifiable. Pathological findings: pap, papillary adenocarcinoma; tub 1, well-differentiated tubular adenocarcinoma; tub 2, moderately differentiated; por 1, poorly differentiated adenocarcinoma (solid type); por 2, (non-solid type); □ sig, signet-ring cell carcinoma; muc, mucinous adenocarcinoma. T; Depth of tumor invasion: T1a, mucosa; T1b, submucosa; T2, mucosa propria; T3, Subserosa; T4a, Serosa exposure; T4b, Serosa invasion. N;Lymph node metastases: N0, no metastases; N1, 1–2 metastases; N2, 3–6 metastases; N3, more than 7 metastases M; Distant metastases: M0, no metastases; M1, metastases. Stage; UICC/TNM 7th edition
